# Semantic Communication: A Survey of Its Theoretical Development

**DOI:** 10.3390/e26020102

**Published:** 2024-01-24

**Authors:** Gangtao Xin, Pingyi Fan, Khaled B. Letaief

**Affiliations:** 1 Department of Electronic Engineering, Tsinghua University, Beijing 100084, China; xgt19@mails.tsinghua.edu.cn; 2 Beijing National Research Center for Information Science and Technology, Tsinghua University, Beijing 100084, China; 3 Department of Electrical and Computer Engineering, Hong Kong University of Science and Technology (HKUST), Hong Kong 999077; eekhaled@ust.hk

**Keywords:** semantic information theory, semantic communication, semantic distortion, 6G, goal-oriented communications, joint source-channel coding, deep learning, information bottleneck

## Abstract

In recent years, semantic communication has received significant attention from both academia and industry, driven by the growing demands for ultra-low latency and high-throughput capabilities in emerging intelligent services. Nonetheless, a comprehensive and effective theoretical framework for semantic communication has yet to be established. In particular, finding the fundamental limits of semantic communication, exploring the capabilities of semantic-aware networks, or utilizing theoretical guidance for deep learning in semantic communication are very important yet still unresolved issues. In general, the mathematical theory of semantic communication and the mathematical representation of semantics are referred to as semantic information theory. In this paper, we introduce the pertinent advancements in semantic information theory. Grounded in the foundational work of Claude Shannon, we present the latest developments in semantic entropy, semantic rate-distortion, and semantic channel capacity. Additionally, we analyze some open problems in semantic information measurement and semantic coding, providing a theoretical basis for the design of a semantic communication system. Furthermore, we carefully review several mathematical theories and tools and evaluate their applicability in the context of semantic communication. Finally, we shed light on the challenges encountered in both semantic communication and semantic information theory.

## 1. Introduction

In recent years, the rapid development of wireless communications and the increasing demand for intelligent processing have given rise to remarkable growth in various emerging intelligent services. However, this surge has brought new challenges to communication and computing technology. On the one hand, the success of these emerging intelligent businesses, such as the industrial Internet, virtual/augmented/mixed reality, metaverse, and holographic communications, heavily relies on training large foundational models with extensive datasets. The substantial traffic generated by these new applications has the potential to overload existing communication networks. Consequently, it is crucial for the communication infrastructure to integrate intelligence, ensuring the efficient and organized handling of traffic in a timely manner. It seems that artificial intelligence technology will trigger and push the rapid developments of semantic information theory in the new era. On the other hand, these intelligent services require extremely low end-to-end latency. For instance, in the realm of autonomous driving, vehicles depend on near-instantaneous data exchange to make split-second decisions, thereby avoiding potential traffic accidents. Similarly, in the context of remote surgery systems, the timely update of surgical tool positions is necessary to ensure the safety and precision of medical procedures. As a result, communication technology must take into account the relevance and urgency of traffic, enabling the swift and reliable extraction and delivery of task-related information. This paradigm shift highlights the importance of the evolution of communication network architecture, shifting from a singular emphasis on high-speed symbol transmission to a prioritization of high-quality semantic exchange [1,2,3].

Semantic communication is a novel architecture that seamlessly integrates tasks and user requirements into the communication process, which is expected to greatly improve communication efficiency and user experience. It emphasizes the efficient exchange of semantics and the clear communication of meaning. Furthermore, this innovative paradigm has the potential to fundamentally address the complex compatibility issues that have plagued traditional data-based communication systems, including challenges spanning cross-system, cross-protocol, and cross-network domains. Around 70 years ago, Weaver [4] categorized communications into three levels:

- Level A: How accurately can the symbols of communication be transmitted? (The technical problem.)
- Level B: How precisely do the transmitted symbols convey the desired meaning? (The semantic problem.)
- Level C: How effectively does the received meaning affect conduct in the desired way? (The effectiveness problem.)

**Figure 1:**
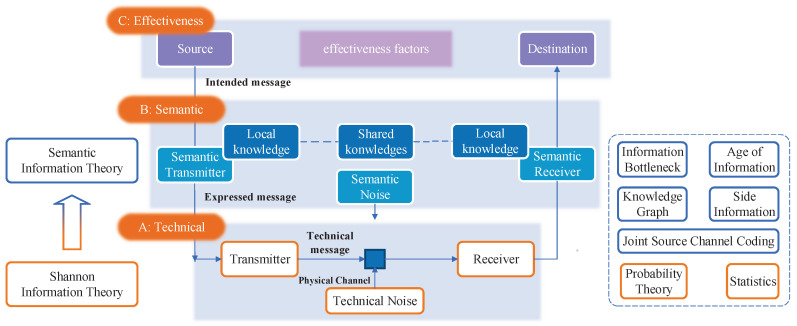
A three-level communication architecture. This includes the technical problem, semantic problem, and effectiveness problem of communication, as well as the mathematical foundation behind Shannon’s information theory and semantic information theory.

Figure 1 illustrates a visual representation of the three-level communication architecture and its underlying mechanism. The communication framework comprises three distinct levels of communication, along with theories and methodologies related to information theory and semantic information theory. At the technical level, a sender transmits a technical message to a receiver through physical channels, thereby introducing technical noise. The primary objective at this level is for the receiver to accurately recover the technical message from the symbols that have been subjected to interference. Moving on to the semantic level, the sender leverages both local and shared knowledge to encode semantics oriented for specific tasks and scenarios. Likewise, the semantic receiver utilizes the knowledge to perform semantic decoding, thereby facilitating the transmission of semantics, which will greatly help to speed up the completion of tasks, or implementation of particular targets. At the effectiveness layer, the concern is whether the received semantics affect conduct as expected in the desired way.

Moving from the bottom to the top of Figure 1, one encounters the technical layer, semantic layer, and effectiveness layer of communication. From the technical layer to the semantic layer, the goal of communication shifts from the accurate transmission of data to the effective exchange of semantics embedded in data. These evolving communication goals necessitate corresponding changes in mathematical theory. The classic Shannon’s information theory, rooted in probability and statistics, primarily addresses the technical layer’s concerns, such as data compression and the communication transmission rate. However, it falls short when applied to the semantic layer since it disregards the semantics of data information and fails to account for crucial semantic factors, such as task relevance, time constraints, and urgency.

In general, the mathematical theory of semantic communication and the mathematical representation of semantics can be attributed to the problem of semantic information theory. Shannon’s information theory primarily relies on tools derived from probability theory and statistics. In contrast, semantic information theory extends its toolkit beyond these fundamentals, incorporating additional methods, such as information bottleneck (IB) and age of information (AoI) to conduct comprehensive research, while the knowledge bases of tasks related will be involved by artificial intelligent tools. Although a recognized and unified theoretical framework for semantic information theory is currently absent, there has been a notable surge in research activities within both academia and industry in recent years. These endeavors have generated a need for the systematic organization and summarization of existing research findings, which can serve as a catalyst for further exploration and advancement in the field of semantic information theory.

Several reviews have introduced the realm of semantic communication. In [5,6,7,8,9,10,11,12,13], they have primarily centered on aspects related to systems, algorithms, and architectures, as well as their connections with deep learning (DL). This article takes a distinctive perspective by focusing on the theoretical dimension—specifically, semantic information theory. From this point, we aim to comprehensively review and examine recent advancements and to chart the future directions of semantic information theory. Grounded in the foundational work of Claude Shannon, we present the latest developments in semantic entropy, semantic rate-distortion, and semantic channel capacity. Moreover, we establish connections between semantic information theory and Shannon’s information theory, with a primary focus on some core concepts of semantic information theory. Furthermore, we introduce various mathematical theories and tools, including concepts like the AoI and IB, which hold significant potential to propel semantic information theory forward.

The rest of this article is structured as follows: Section 2 provides an introduction to semantics and semantic communication. Section 3, Section 4 and Section 5 constitute an in-depth exploration of the fundamental concepts within semantic information theory. These sections cover essential topics, including semantic entropy, semantic rate-distortion, and semantic channel capacity. In Section 6, we introduce the mathematical theories and tools that are relevant to the domain of semantic communication. Section 7 discusses the potential challenges that may arise during the development of semantic communication and semantic information theory. Finally, we conclude the paper in Section 8.

## 2. Semantic Communication

Although this paper serves as a summary of the latest research advancements in semantic information theory, it is necessary to establish an intuitive understanding of semantics and semantic communication before considering the theoretical part. In this section, we present a brief introduction to semantic communication and introduce a general semantic communication system.

### 2.1. What Is Semantic Communication?

Semantic communication serves distinct motivations and purposes compared to traditional digital communication. In Shannon’s landmark 1948 paper, Shannon [14] stated that

*The fundamental problem of communication is that of reproducing at one point either exactly or approximately a message selected at another point.*

While Weaver [4] emphasized that

*The semantic problems are concerned with the interpretation of meaning by the receiver, as compared with the intended meaning of the sender.*

These two statements correspond to different levels of communication. Shannon’s sentence addresses the technical layer, specifically digital communication, while Weaver’s sentence focuses on the semantic layer, namely, semantic communication. By comparing these two statements, we can find that the objective of semantic communication is not to replicate the transmitted messages, whether exact or approximate, but rather to convey their interpretations accurately. For example, consider the following conversation:

Alice: “Do you like bananas?”

Bob: “No, I hate eating any fruit.”

In this conversation, Alice serves as a semantic sender while Bob assumes the role of a semantic receiver. Bob is able to interpret the meanings of the received message and relate it to his existing vocabulary. He knows that “hate” is an antonym of “like”, and “banana” falls under the category of “fruit”. Consequently, he can infer that “hate eating any fruit” implies “do not like bananas”, despite the fact that the two statements have distinct syntactical structures [15]. Now, consider a conversation between three persons:

Alice: “Bob, does Carol like bananas?”

Bob: “Carol, if you enjoy bananas?”

Carol: “No, I do not enjoy any fruit.”

In this context, Bob acts as a semantic channel between Alice and Carol. While Bob may not precisely replicate Alice’s original message, he adeptly retains its intended meaning. While assessing the success of communication in a purely literal sense, there might be an engineering failure. However, there is no failure at the semantic level.

From these two examples, we can see that the objective of semantic communication lies in the effective exchange of semantics. In other words, whether the meaning carried by the symbol can be understood by the receiver. This model of communication capitalizes on the participants’ perception of the world and their responses to various concepts, thereby giving symbols a deeper and more abstract connotation. In summary, semantic communication is not to reproduce, but to convey after understanding. In the aforementioned examples, semantics is explored within a conversational context. Over 70 years of development, the concept of semantics has evolved beyond its initial confines within language. It now extends its reach into diverse dimensions, encompassing images, videos, audio, and more. Semantics has become intricately connected to specific scenarios and tasks, and is adept at extracting features that align more closely with the requirements of the specific task.

### 2.2. What Is a Semantic Communication System?

In general, current semantic communication systems are constructed upon digital communication frameworks. In other words, these systems still depend on the physical channel to transmit specific symbols and are not entirely detached from Shannon’s paradigm, which is consistent with Weaver’s viewpoint. A semantic communication system usually comprises the following key components [7]:

- Semantic Encoder: This component is responsible for detecting and extracting the semantic content from the source message. It may also compress or eliminate irrelevant information to enhance efficiency.
- Channel Encoder: The role of the channel encoder is to encode and modulate the semantic features of the message as signals to combat any noise or interference that may occur during transmission.
- Channel Decoder: Upon receiving the signal, the channel decoder demodulates and decodes it, recovering the transmitted semantic features.
- Semantic Decoder: The semantic decoder interprets the information sent by the source and converts the received signal features into a format that is comprehensible to the destination user.
- Knowledge Base: The knowledge base serves as a foundation for the semantic encoder and decoder, enabling them to understand and infer semantic information accurately and effectively.

**Figure 2:**
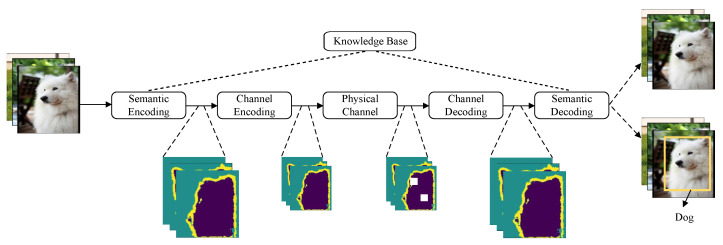
A general semantic communication architecture [5]. It is oriented to the image recognition task and only transmits task-relevant feature content.

In general, a semantic communication system includes the five mentioned components, with the flexibility to add more as required for specific tasks. Figure 2 illustrates a general semantic communication architecture for the transmission task of image recognition. Rather than transmitting bit sequences that represent the entire image, the semantic transmitter in this architecture extracts only the features crucial for recognizing the object—in this case, a dog—from the source. Irrelevant information, like the image background, is intentionally omitted to minimize the transmitted data while maintaining performance quality [5]. In this model, the knowledge base empowers the semantic encoder and decoder to generate and reconstruct semantics related to image recognition, respectively.

In summary, a semantic communication system is not a hypothetical concept but is built on a digital communication system, as it relies on physical channels to transmit essential symbols. The information carried by symbols results from the empowerment of the knowledge base. In the following section, we introduce several important concepts and theorems within semantic information theory, while also highlighting its distinctions from classical information theory.

## 3. Semantic Entropy

Entropy, which measures the uncertainty of a random variable, constitutes a fundamental concept in Shannon’s information theory. Likewise, the quantification of semantic information forms the cornerstone of semantic information theory, referred to as semantic entropy. Semantic entropy serves as a metric for quantifying semantic uncertainty or the amount of semantic information. However, formulating an intuitive and universally applicable mathematical description of semantic entropy remains a formidable task. On the one hand, the semantic connotation is elusive to define and quantify. On the other hand, the generation mechanisms and processes of semantics remain obscure [3,8,16]. In this section, we introduce the essence of semantics and examine various definitions of semantic entropy.

### 3.1. Statistical and Logical Probability

Let *X* be a discrete random variable with alphabet X and probability mass function p(x)=Pr{X=x},x∈X. In Shannon’s information theory, the entropy H(X) of a discrete random variable *X* is defined by

(1) $$H\left(X\right)=-\sum_{x\in X}p\left(x\right)\log p\left(x\right).$$

In Equation (1), p(x) is the statistical probability of *x*, reflecting its frequency information. However, statistical probability is no longer the exclusive mathematical tool of choice for semantic communication. In general, probability includes two aspects, one is about logical probability and the other is about statistical probability [17].

- Logical Probability: Logical probability pertains to the degree of confirmation of a hypothesis with respect to an evidence statement, such as an observation report. A sentence regarding this concept relies on logical analysis rather than the direct observation of facts.
- Statistical Probability: Statistical probability refers to the relative frequency (in the long run) of one property of events or things with respect to another. A sentence concerning this concept is grounded in factual and empirical observations.

Shannon chose statistical probability as the mathematical foundation of information theory due to its ability to leverage principles from probability theory and stochastic processes. Through the application of the law of large numbers, Shannon can derive an asymptotic equipartition property, paving the way for the derivation of several key theorems in information theory. Nonetheless, when it comes to semantic entropy, there is no widely accepted consensus on whether to employ statistical probability or logical probability. In the following discussion, we will see that semantic entropy was initially formulated using logical probability and subsequently evolved into various distinct formulations.

### 3.2. Semantic Entropy

Semantic information represents the semantic features carried by a message or symbol within specific scenarios and tasks. It is tailored to particular contexts. Semantic entropy serves as a tool for quantifying semantic information including feature representation and knowledge distillation. Consequently, semantic entropy can be computed, manipulated, and compared for analytical purposes. Semantic entropy originates from the analysis of natural language. In 1952, Carnap and Bar-Hillel [18] proposed the concept of semantic entropy as a means to quantify the amount of semantic information conveyed by a sentence. This concept aimed to assess the depth of meaning within a sentence, capturing its richness in conveying information. Let m(e) be the logical probability of event *e*, which signifies the likelihood that the sentence holds true in all possible situations. Then, the semantic entropy $H_{s}\left(e\right)$ is defined by

(2) $$H_{s}\left(e\right)=-\log m\left(e\right).$$

It is evident that the higher the logical probability of a sentence, the lower the semantic entropy. However, this gives rise to a paradox. Any statement that contradicts itself will possess an infinite amount of semantic information, such as $H_{s}\left(e,\;\neg e\right)$ (“¬e” represents the counter-event or complementary event of “*e*”), which becomes infinite.

In 2004, Floridi [19] proposed the strong semantic information theory, which resolved this paradox by utilizing the distance from actual events to represent the quantity of information. In 2011, D’Alfonso [20] provided a quantitative description of semantic information based on truthlikeness. Both Floridi and D’Alfonso measured the semantic information of a particular event relative to a reference event, yielding a value ranging from 0 to 1. However, these measurements heavily depend on the existence of reference events. Without a reference event, it becomes impossible for them to quantify the semantic entropy. Essentially, their work provides a measure of semantic similarity between two sentences, rather than a gauge of semantic uncertainty or informativeness. In alignment with Carnap’s definition, several works have enriched and provided a more specific representation of semantic entropy by extending the connotation of m(e) [21]. In 2011, Bao [15] used propositional logic to expand the representation of m(e). For a message (sentence) *e*, let $W_{e}$ be the set of its models, i.e., worlds in which *x* is “true”, $W_{x}=\left\{w\in W|w⊢x\right\}$ (⊢ is the logical entailment relation). Let μ(w) be the statistical probability of model *w*. Then, the logical probability of *e* is

(3) $$m\left(e\right)=\frac{\mu (W_{e})}{\mu (W)}=\frac{\sum_{w\in W,w⊢e}\mu \left(w\right)}{\sum_{w\in W}\mu \left(w\right)}.$$

Equation (3) shows that what matters is the total probability of the sentence model, not the model set’s cardinality. With the development of technologies such as artificial intelligence, scholars have a new understanding of semantics. The scope of semantics is no longer limited to sentences but also has new connotations in images, videos, audio, etc., especially for feature representations based on knowledge bases. Semantics is oriented to specific scenarios, extracting features that align more seamlessly with the demands of the present task. In addition to the aforementioned research for sentences, we classify the work on semantic entropy into several distinct categories. Moreover, some of these methods are not limited to probability theory, attempting to define semantic entropy using other mathematical theories.

(1) Task-oriented: The meaning and mechanism of semantic entropy should have various representations to suit different tasks. Chattopadhyay et al. [22] proposed the quantification of task-related semantic entropy, defined as the minimum number of semantic queries about data *X* required to solve the task *V*. It can be expressed as

(4) $$\begin{array}{c} H_{s}\left(X;V\right)≜\min_{E}E[|{\mathrm{Code}}_{Q}^{E}\left(X\right)\left|\right], \end{array}$$

where ${\mathrm{Code}}_{Q}^{E}\left(x\right)$ represents the query vector extracted from *x* using semantic encoder *E*.

For translation tasks, Melamed [23] proposed a method to measure the semantic entropy of words in a text. Specifically, let *w* represent a given word, the semantic entropy can be expressed as

(5) $$H_{s}\left(w\right)=H\left(T|w\right)+N\left(w\right)=\sum_{t\in T}p\left(t|w\right)\log p\left(t|w\right)+p\left(\mathrm{Null}|w\right)\log F\left(w\right).$$

Among these components, p(t|w) is the statistical transition probability, H(T|w) represents the translation inconsistency, signifying the uncertainty when translating a word, where *T* represents the set of target words. N(w) reflects the impact of empty links for word *w*, indicating the likelihood of encountering translation difficulties between languages. F(w) represents the frequency of word *w*, and p(Null|w) is the probability of encountering problems when translating *w*.

For classification tasks, Liu et al. [24] introduced the concepts of matching degree and membership degree to define semantic entropy. Membership degree, a concept from fuzzy set theory [25], is challenging to express analytically. It is generally given based on experience. If we denote $\mu_{\varsigma }\left(x\right)$ as the membership degree of each x∈X, then for a specific category $C_{j}$, the matching degree is defined as

(6) $$D_{j}\left(\varsigma \right)=\frac{\sum_{x\in X_{C_{j}}}\mu_{\varsigma }\left(x\right)}{\sum_{x\in X}\mu_{\varsigma }\left(x\right)}.$$

For category $C_{j}$, its semantic entropy is defined as $H_{C_{j}}\left(\varsigma \right)=-D_{j}\left(\varsigma \right)\log D_{j}\left(\varsigma \right)$. To obtain the overall semantic entropy for *X*, one can sum the semantic entropy contributions from all categories, which is expressed as

(7) $$H_{s}\left(\varsigma \right)=\sum_{j}H_{C_{j}}\left(\varsigma \right).$$

(2) Knowledge-based: Semantics involves the comprehension of symbols, and knowledge plays a crucial role in the process of semantic encoding and representation. Choi et al. [26] explored the semantic entropy of a sentence from the perspective of knowledge bases using logical probability. Let the knowledge base be denoted as *K*. Let m[K⊢e] be the probability that *e* is true relative to the knowledge base *K*, which can be simplified as $m_{e}$. Then, the semantic entropy of *e* relative to *K* is calculated as:

(8) $$H_{s}\left(e\right)=-m_{e}\log m_{e}+\left(1-m_{e}\right)\log\left(1-m_{e}\right),$$

which quantifies the semantic entropy of *e* with respect to the knowledge base *K*.

Moreover, expansion has the capability to amalgamate simple elements into complex systems, potentially leading to the emergence of intelligence. In the realm of human language, sentences are constructed from components such as subjects, predicates, objects, and attributive complements, enabling the expression of profound meanings that single words cannot convey. Xin and Fan [27,28] advocated for the extensibility of semantics, emphasizing that the representation of semantic entropy should encompass the notion of expansion. As semantics expand, knowledge often involves collisions. This can be likened to the phenomenon where as a country expands its territory, armed conflicts may arise. Semantics is a product born from the interaction between knowledge and signals. For instance, while “Apple Inc.” falls under the category of a business company, and “fifteenth” is a numerical concept, their collision can give rise to a new word—“iPhone”—signifying a mobile communication product. Let $X_{1}$ and $X_{2}$ denote signals, and let $K_{A}^{1}$ and $K_{A}^{2}$ represent two instances of knowledge. Let *T* and $\hat{T}$ be the semantics of the transmitter and receiver, respectively. Then, one step of the expansion architecture of semantic communication is described:

(9) $$\begin{array}{cc} \; & X\;\;\;\;\;\;\;\overset{\left(a\right)}{⟶}\;\;\;\;\;\;\;\hat{X}\\ \; & ⇑\;\;\;\;\;\;\;\;\;\;\;\;\;\;\;\;\;\;⇓\\ T\;\leftarrow \;\;X_{1} & ⊕X_{2}\;\;\;\;\;\;\;\;\;\;\;\;{\hat{X}}_{1}⊕{\hat{X}}_{2}\;\to \;\hat{T}\\ \; & ↑\begin{array}{c} \left(c\right) \end{array}\;\;\;\;\;\;\;\;\;\;\;\;\;\;\;\;↑\begin{array}{c} \left(d\right) \end{array}\\ K_{A}^{1} & ⊙K_{A}^{2}\;\;\;\overset{\left(b\right)}{⤏}\;\;K_{B}^{1}⊙K_{B}^{2} \end{array}$$

where (a) is the explicit channel, (b) is the implicit channel, and (c) and (d) are the semantic encoding and decoding, respectively. The semantic entropy can be expressed as $H_{s}\left(X_{1}⊕X_{2},K_{A}^{1}⊗K_{A}^{2}\right)$, where ⊕ denotes expansion and ⊗ represents collision.

(3) Context-related: The forms of derivation for semantic entropy also vary depending on the specific context. Kountouris and Pappas [8] defined a context-dependent entropy as

(10) $$H_{S}\left(P\right)=-\sum_{x\in X}\varphi \left(x\right)P\left(x\right)\log P\left(x\right),$$

where *P* is a statistical probability mass function on a discrete set X. Additionally, φ(·) is a function that weights the different outcomes with respect to their utility for a specific goal. Moreover, Kowlchinsky and Wolpert [29] defined semantic information as grammatical information that describes the relationship between a system and its environment. Venhuizen et al. [30] derived semantic entropy from a language understanding model grounded in background knowledge. Lu [31] introduced general information theory and employed concepts such as the Bayesian formula, logical probability, and fuzzy sets to mathematically describe semantic information.

## 4. Semantic Rate Distortion

In the communication process, achieving a perfect performance is not always possible. It is conceivable for the receiver to obtain symbols that do not align with those sent by the sender. Additionally, describing an arbitrary real number necessitates an infinite number of bits. Therefore, representing a continuous random variable with a finite representation can never be flawless. To approach this question appropriately, it is necessary to establish the quality of a source’s representation. This is achieved by defining a distortion measure, which serves as a metric for evaluating the disparity between the random variable and its representation. In Shannon’s information theory, rate-distortion theory addresses the coding problem of continuous random variables or vectors in the presence of distortion [32].

If we possess a source capable of generating a sequence $X_{1},X_{2},\ldots ,X_{n},\;i.i.d.∼p\left(x\right),x\in X$. The encoder describes the source sequence $X^{n}$ by an index $f_{n}\left(X^{n}\right)\in \left\{1,2,\ldots ,2^{nR}\right\}$. The decoder represents $X^{n}$ by an estimate ${\hat{X}}^{n}\in {\hat{X}}^{n}$. A distortion function, or distortion measure, is a mathematical mapping denoted as $d:X\times \hat{X}\to R^{+}$. It operates on pairs of elements, each drawn from the source alphabet X and the reproduction alphabet $\hat{X}$, and produces non-negative real numbers as its output. The distortion, denoted as $d(x,\hat{x})$, quantifies the cost associated with representing the symbol *x* using the symbol $\hat{x}$. When considering sequences, such as $x^{n}$ and ${\hat{x}}^{n}$, the distortion $d(x^{n},{\hat{x}}^{n})$ is calculated as the average per-symbol distortion across the elements of the sequence. Based on this, one can define the rate-distortion function.

**Definition** **1.** *The rate-distortion function for a source X∼p(x) and distortion measure $d(x,\hat{x})$ is*

(11) $$R\left(D\right)=\min_{p(\hat{x}|x)}I\left(X;\hat{X}\right),$$

*where the minimization is over all conditional contributions $p(\hat{x}|x)$ for which the joint distribution $p\left(x,\hat{x}\right)=p\left(x\right)p\left(\hat{x}|x\right)$ satisfies the expected distortion constraint $E(d\left(X,\hat{X}\right))\leq D$.*

The value R(D) represents the infimum of rates *R* achievable for a given distortion *D*. Building upon the foundation of the rate-distortion function, Shannon subsequently derived the influential rate-distortion theorem.

**Theorem** **1.** *(Rate-distortion theorem) If R>R(D), there exists a sequence of codes ${\hat{X}}^{n}\left(X^{n}\right)$ with the number of codewords $|{\hat{X}}^{n}\left(\cdot \right)|\leq 2^{nR}$ and $E\;d(X^{n},{\hat{X}}^{n}\left(X^{n}\right))\to D$. If R≤R(D), no such codes exist.*

The rate-distortion theorem addresses two fundamental results regarding distortions: Firstly, given a source distribution and a distortion measure, it determines the minimum expected distortion achievable at a specific rate. Secondly, it establishes the minimum rate description required to achieve a particular distortion. In this section, we introduce the semantic level of rate-distortion theory, drawing upon Shannon’s foundation work. We explore topics such as semantic mismatch, the semantic rate-distortion theorem, and semantic coding.

In the field of semantic information theory, the exploration of semantic rate-distortion holds significant importance. In related studies, two distortion measurement functions, namely, semantic distortion and symbol distortion, are commonly employed to assess the effects of coding on communication quality. Utilizing established semantic evaluation criteria, defining and implementing semantic rate-distortion becomes more accessible when compared to the complexities of semantic entropy and semantic channel capacity. Next, we first introduce the semantic mismatch evaluation criteria for various objects.

### 4.1. Metrics for Semantic Mismatch

In general, the quality of semantic communication diverges from the traditional measure of the bit error rate (BER) and the symbol error rate (SER) commonly used in digital communication. Semantic communication often employs metrics capable of assessing semantic similarity, which aligns more closely with human perception. Furthermore, there is no universal metric for semantic mismatch. One-size-fits-all is unrealistic and impossible in semantic communication. It generally adapts to specific tasks for information sources. In this work, we will concentrate on a select set of representative metrics. We begin by introducing the performance metrics employed in contemporary semantic communication systems for images, text, and audio, respectively.

(1) Image: The measurement of similarity between two images, denoted as *A* and *B*, is expressed as follows:

(12) $$L\left(A,B\right)={∥f\left(A\right)-f\left(B\right)∥}_{2}^{2},$$

where f(·) represents the image embedding function, which maps an image to a point in Euclidean space, as outlined in [5]. While the peak signal-to-noise ratio (PSNR) and the structural similarity index (SSIM) serve as common image metrics, it is necessary to note that these metrics primarily operate at the per-pixel level, failing to capture differences in semantics and human perception.

DL-based image similarity metrics have the capacity to capture semantics to a certain extent. Johnson et al. [33] introduced two concepts known as perceptual loss functions, which enable the measurement of high-level perceptual and semantic distinctions between images. These perceptual loss functions are constructed using a loss network denoted as ϕ, which is pre-trained for image classification. It is worth noting that these perceptual loss functions themselves are deep convolutional neural networks. The perceptual loss functions consist of a feature reconstruction loss and a style reconstruction loss. Let $\phi_{j}\left(A\right)$ be the activations of the *j*-th layer of the network ϕ when processing the image *A*, and let *L* represent the shape. Then, the feature reconstruction loss is the Euclidean distance between feature representations:

(13) $$L_{\mathrm{feat}}\left(A,B\right)=\frac{1}{L}{∥\phi \left(A\right)-\phi \left(B\right)∥}_{2}^{2}.$$

The style reconstruction loss is responsible for capturing the stylistic characteristic of images. It is defined as the squared Frobenius norm of the difference between the Gram matrices, $G_{l}^{\phi }$, of two images, and it is expressed as follows:

(14) $$L_{\mathrm{style}}\left(A,B\right)={∥G_{l}^{\phi }\left(A\right)-G_{l}^{\phi }\left(B\right)∥}_{F}^{2}.$$

Deep features have proven to be highly effective in semantic tasks and serve as robust models for understanding human perceptual behavior. Notably, Zhang et al. [34] conducted a comprehensive evaluation of deep features across various architectural designs and tasks. Their research compared these deep features with traditional metrics, and the results demonstrated a significant superiority of deep features. They outperformed previous metrics by substantial margins, particularly on a newly introduced dataset focused on human perceptual similarity judgments.

In a related development, Wang et al. [35] proposed a deep ranking model designed to learn fine-grained image similarity models. It utilizes a triplet-based hinge loss ranking function to characterize fine-grained image similarity relationships. It also incorporates a multiscale neural network architecture capable of capturing both global visual properties and image semantics. Additionally, in 2023, Zhu et al. [36] proposed ViTScore, a novel semantic similarity evaluation metric for images. ViTScore relies on the pre-trained image model ViT (Vision Transformer) and represents a cutting-edge approach to assessing semantic similarity in the context of images.

(2) Text: In the context of text transmission, conventional metrics, such as the word-error rate (WER), often struggle to effectively address semantic tasks, as pointed out by Farsad et al. [37]. In response to this challenge, several metrics based on semantics have been proposed to reflect the dissimilarity of word meanings, such as the semantic error measure [38]. Specifically, the bilingual evaluation understudy (BLEU) metric, initially designed for machine translation evaluations by Papineni et al. [39], has found utility in the domain of semantic communication. BLEU assesses the quality of semantic communication as follows: Let $l_{a}$ and $l_{b}$ represent the word lengths of sentences *a* and *b*, respectively, then the BLEU score is defined by

(15) $$\log\mathrm{BLEU}=\min\left(1-\frac{l_{a}}{l_{b}},0\right)+\sum_{n=1}^{N}w_{n}\log p_{n},$$

where $w_{n}$ is the weight of the *n*-grams, and $p_{n}$ denotes the *n*-grams score, which is defined as

(16) $$p_{n}=\frac{\sum_{k}\min\left(C_{k}\left(\hat{s},C_{k}\left(s\right)\right)\right)}{\sum_{k}\min\left(C_{k}\left(\hat{s}\right)\right)},$$

where $C_{k}\left(\cdot \right)$ is the frequency count function for the *k*-th element in the *n*-th gram.

The concept of sentence similarity, as proposed in [40], serves as a metric for quantifying the semantic similarity between two sentences. It is expressed as:

(17) $$\tau \left(\hat{s},s\right)=\frac{B_{\Phi }\left(s\right)\cdot B_{\Phi }{\left(\hat{s}\right)}^{T}}{∥B_{\Phi }\left(s\right)∥∥B_{\Phi }\left(\hat{s}\right)∥}.$$

where $B_{\Phi }\left(\cdot \right)$ represents the BERT model [41], which maps a sentence to its semantic vector space. This model is pre-trained on a massive dataset comprising billions of sentences, enabling it to capture rich semantic information.

(3) Audio: In the realm of semantic communication, novel perception-based audio metrics are employed, including the perceptual evaluation of speech quality (PESQ) [42], the short-time objective intelligibility (STOI) [43], and the unconditional Frechet deep speech distance (FDSD) [44], etc. These metrics provide valuable insights into the semantic aspects of audio quality and perception. In general, these metrics assess the similarity between two audios at a semantic or higher-dimensional feature level. For example, given the samples *X* and *Y*, the FDSD is defined as

(18) $$L_{FDSD}={∥\mu_{X}-\mu_{Y}∥}_{2}^{2}+Tr\left(\Sigma_{X}+\Sigma_{Y}-2{\left(\Sigma_{X}\Sigma_{Y}\right)}^{1/2}\right),$$

where $\mu_{X}$, $\mu_{Y}$ and $\Sigma_{X}$, $\Sigma_{Y}$ are the means and covariance matrices of *X* and *Y*, respectively.

### 4.2. Semantic Rate-Distortion Theorem

In the context of semantic communication, the process of feature extraction and coding representation at the semantic level plays a crucial role in reducing information redundancy and extracting the most salient semantic features, thus improving the effectiveness of semantic transmission. The semantic rate-distortion theorem is a theoretical framework designed to address the challenges associated with distortion and encoding in semantic communication. It offers solutions and insights into optimizing the trade-off between preserving semantic content and achieving efficient encoding. Liu et al. [45] introduced a comprehensive semantic rate-distortion theory framework. In this framework, they consider a memoryless information source represented as a tuple of random variables, denoted as (S,X). It has a joint probability distribution denoted as p(s,x) within a product alphabet S×X. Here, *S* represents the intrinsic state, capturing the “semantic” aspect of the source, which is not directly observable. On the other hand, *X* represents the extrinsic observation of the source, capturing the “appearance” as perceived by an observer.

**Figure 3:**
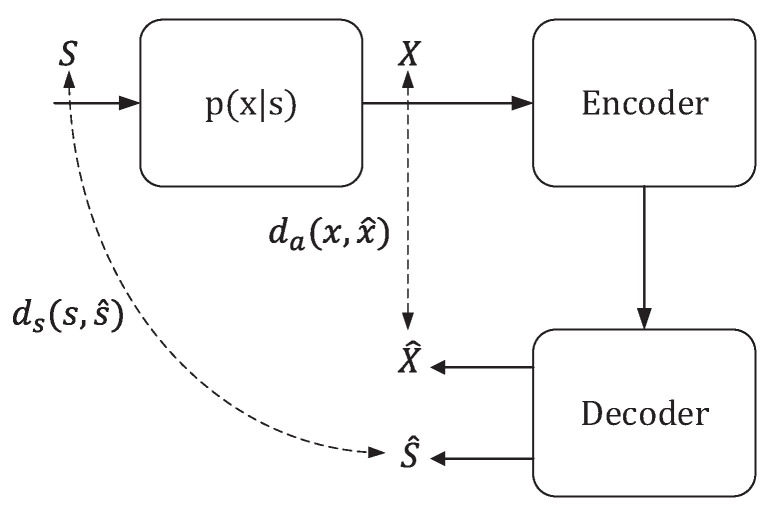
A semantic communication framework for introducing semantic rate-distortion [45]. In this framework, two distortion metrics are considered: $d_{s}\left(s,\hat{s}\right)$ representing semantic distortion, and $d_{a}\left(x,\hat{x}\right)$ representing appearance distortion.

For a length-*n* independent and identically distributed (i.i.d.) sequence from the source, denoted as $\left(S^{n},X^{n}\right)$, a source encoder $f_{n}$ with a rate of *R* is a mapping that transforms $X^{n}$ into an index within the set $\left\{1,2,\ldots ,2^{nR}\right\}$. This encoder corresponds to a decoder that maps the index back into a pair of sequences, denoted as $\left({\hat{S}}^{n},{\hat{X}}^{n}\right)$, where these sequences are drawn from the product alphabet $\hat{S}\times \hat{X}$. This process is illustrated in Figure 3.

In this framework, two distortion metrics are considered: $d_{s}\left(s,\hat{s}\right)$ representing the semantic distortion, and $d_{a}\left(x,\hat{x}\right)$ representing the appearance distortion. These metrics map elements from alphabets $S\times \hat{S}$ and $X\times \hat{X}$ to non-negative real numbers. Consequently, the block-wise distortions are defined as:

(19) $$\begin{array}{cc} d_{s}\left(s^{n},{\hat{s}}^{n}\right) & =\frac{1}{n}\sum_{i=1}^{n}d_{s}\left(s_{i},{\hat{s}}_{i}\right), \end{array}$$

(20) $$\begin{array}{cc} d_{a}\left(x^{n},{\hat{x}}^{n}\right) & =\frac{1}{n}\sum_{i=1}^{n}d_{a}\left(x_{i},{\hat{x}}_{i}\right). \end{array}$$

Moreover, the framework defines the semantic rate-distortion function as

(21) $$\begin{array}{cc} R(D_{S},D_{a}) & =\min I(X;\hat{S},\hat{X}), \end{array}$$

(22) $$\begin{array}{cc} s.t.\;\;E\;{\hat{d}}_{s}\left(X,\hat{S}\right) & \leq D_{s},\;\;E\;{\hat{d}}_{a}\left(X,\hat{X}\right)\leq D_{a} \end{array}$$

where *S* and $\hat{S}$ represent the semantic understanding of the sender and the receiver, while *X* and $\hat{X}$ are their respective semantic representations. Expanding on this, Guo et al. [46] proposed the analysis of semantic rate-distortion involving two users, considering the perspective of side information. This perspective can be expressed as:

(23) $$R\left(D_{1},D_{2},D_{s}\right)=\min I\left(X_{1},X_{2};{\hat{X}}_{1},{\hat{X}}_{2},\hat{S}|Y\right),$$

where $X_{1}$ and $X_{2}$ are the semantic representations of two users, respectively. *Y* represents the side information. In 2022, Stavrou and Kountouris [47] further studied the characteristics of this system, particularly focusing on the Hamming distortion metric.

### 4.3. Semantic Coding

Semantic rate-distortion theory directly corresponds to coding technology. For a given transmitting task, a semantic coding strategy needs to achieve two potentially conflicting goals:

- Maximizing the expected faithfulness (minimizing expected semantic distortion).
- Minimizing the expected coding length.

An ideal semantic coding strategy should simultaneously minimize both the expected semantic distortion and the expected coding length. However, achieving this delicate balance is highly complex and challenging. In current practice, a common approach involves the use of a dual distortion metric to represent the semantic coding. Shao et al. [48] used the semantic distortion and the semantic cost to define the achievable region for semantic coding. The semantic distortion reflects the disparities in semantic understanding between the receiver and the sender. The semantic cost, which quantifies the simplicity or understandability of information, is often represented as the length of the corresponding bit sequence. The definition of the achievable distortion-cost regions can be expressed as: A distortion-cost pair (L,D) is achievable if there exists a semantic encoding scheme *U* if $D_{U}=D$, $L_{U}=L$.

Agheli [49] et al. explored semantic coding within a multi-user context. They introduced an approach where observations from an information source are filtered and sent to two monitors depending on their importance for each user’s specific objectives. By optimizing the codeword lengths using semantics-aware utility functions, substantial reductions in the amount of communicated status updates can be achieved. Xiao et al. [50] proposed the rate-distortion theory of strategic semantic communication. Their approach integrates game theory models with rate-distortion theory to characterize how information interaction between semantic encoders and decoders impacts communication distortion performance. Furthermore, Tang et al. [51] considered a semantic source that consists of a set of correlated random variables whose joint probabilistic distribution can be described by a Bayesian network. Their work focuses on characterizing the limits of lossy compression for semantic sources and establishing upper and lower bounds for the rate-distortion function.

## 5. Semantic Channel Coding

Channel capacity is the most successful and central contribution to Shannon’s information theory. On the one hand, it provides the maximum number of distinguishable signals through repeated use of a communication channel. By appropriately mapping the source into “widely spaced” input sequences for the channel, one can transmit a message with an exceedingly low probability of error, subsequently reconstructing the source message at the output. On the other hand, the channel capacity represents the rate at which reliable information can be transmitted through a noisy channel, as discussed by Verdú in ‘Fifty Years of Shannon Theory’ [52].

Similarly, the issue of capacity holds immense significance within the realm of semantic communication. In this section, we introduce the concepts of semantic noise and semantic channel capacity. Furthermore, several widely considered questions about semantic channel capacity are raised and addressed. Finally, we attempt to give a general description of the semantic channel capacity.

In the domain of digital communications, a discrete channel is defined as a system comprising an input alphabet denoted by X, an output alphabet represented by Y, and a probability transition matrix p(y|x) that quantifies the probability of observing the output symbol *y* when transmitting the message *x*.

**Definition** **2.** *The channel capacity of a discrete memoryless channel is defined as*

(24) $$C=\max_{p(x)}I\left(X;Y\right),$$

*where the maximum is taken over all possible input distributions p(x) provided by the channel transition probability function p(y|x).*

For the sake of convenient comparison, we commonly refer to this as the physical channel capacity. Moreover, Shannon’s theorem established that information can be transmitted reliably through a physical channel at any rate up to the channel capacity, known as the channel coding theorem.

**Theorem** **2.** *(Channel coding theorem) For a discrete memoryless channel, all rates below the capacity C are achievable. Specifically, for every rate R<C, there exists a sequence of $\left(2^{nR},n\right)$ codes with a maximum probability of error $\lambda^{\left(n\right)}\to 0$, as n→∞. Conversely, any sequence of $\left(2^{nR},n\right)$ codes with $\lambda^{\left(n\right)}\to 0$, as n→∞, must have R≤C.*

The channel coding theorem states that all rates below the capacity *C* are achievable, while rates exceeding this capacity are unachievable. This leads us to contemplate the significance and formulation of the channel capacity in the context of semantic communication, a topic of interest among scholars in the field. In the exploration of semantic information theory, we try to address the following three fundamental questions regarding capacity.

1. Is there an analogous concept of channel capacity in semantic communication, which we may term the ’semantic channel capacity’?
2. Is it possible that the semantic channel capacity is greater than the physical channel capacity?
3. Is there a universal expression for the semantic channel capacity?

Next, we will address these three fundamental questions and introduce the semantic noise along with the semantic channel capacity theorem.

### 5.1. Semantic Noise

Noise introduces uncertainty into communication systems and also poses challenges to communication technologies. In the absence of noise, the transmission and exchange of any information are perfect and lossless, rendering capacity a meaningless concept. Generally, semantic noise exists widely in semantic communication. Prior to the formal introduction, it is essential to clarify that semantic noise and semantic channel noise are distinct concepts. In most of the scholarly literature, semantic noise refers to the mismatch of semantics. The semantic channel noise commonly refers to the discrepancies in the knowledge background of both parties in semantic communication. It is worth noting that semantic noise may be added either at the physical channel or at the semantic channel.

In their respective works, Qin et al. [5], Shi et al. [7], and Hu et al. [53] offered similar definitions of semantic noise within the context of semantic communication. Qin et al. defined semantic noise as a disturbance that affects the interpretation of a message, characterized by a semantic information mismatch between the transmitter and receiver. Similarly, Shi et al. described semantic noise as noise introduced during the communication process, leading to misunderstanding and incorrect reception of semantic information. It can be introduced at various stages, including encoding, data transportation, and decoding. Hu et al. defined semantic noise as a unique form of noise in semantic communication systems, denoting the misalignment between intended semantic symbols and received ones.

Semantic noise varies across different source categories. Semantic noise in text refers to semantic ambiguity, which slightly changes the semantic meaning of a sentence. In the case of images, it can be modeled by using adversarial samples. The following examples illustrate instances of semantic ambiguity in the text, categorized by communication channel [15]:

- The meaning of a message is changed due to transmission errors, e.g., from “contend’’ to “content” (Physical channel)
- Translation of one natural language into another language where some concepts in the two languages have no precise match (Semantic channel)
- Communicating parties use different background knowledge to understand the message (e.g., Apple has different meanings in vegetable markets and mobile phone stores) (Semantic channel)

**Figure 4:**
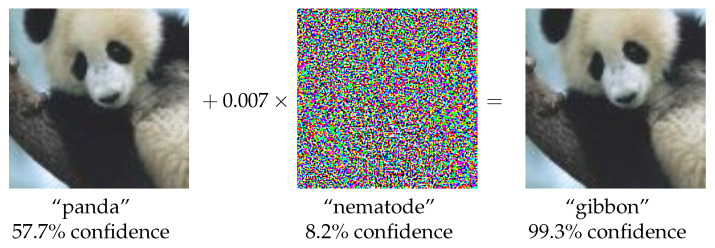
An example of the adversarial sample in the image [54].

An illustrative example of an adversarial image is presented in Figure 4, in which the adversarial samples are added. It is apparent that the image when perturbed with adversarial noise, can mislead DL models in their classification, while remaining visually indistinguishable from the original image to human observers [54].

In summary, semantic noise encompasses both semantic channel noise and physical channel noise. It represents a discrepancy in semantic information between the transmitter and the receiver within the context of semantic communication.

### 5.2. Semantic Capacity

For the first question in Section 5, we argue that determining the capacity of the semantic channel is a challenging task, and expressing it using current mathematical tools remains elusive. On the one hand, to the best of our knowledge, it is still an open problem to effectively model the local knowledge and global knowledge shared by both communicating parties. On the other hand, quantifying and representing the semantic channel noise is also difficult. However, there are some studies that introduce semantic capacity. We would like to clarify that the semantic channel capacity studied in most current works is the capacity at the semantic level, rather than the capacity of the semantic channel. In the subsequent sections of this paper, the semantic channel capacity we refer to is also the information capacity at the semantic level.

In 2016, Okamoto [55] argued that the semantic channel capacity represents the maximum rate of semantic information that can be transmitted over the semantic channel, or the ratio of the maximum semantic communication amount to the communication data size. Similarly, Bao et al. [15] defined the semantic channel capacity as the capacity limit such that a transmission rate can be achieved with arbitrarily small semantic errors within the limit. Specifically, they derived the semantic channel coding theorem.

**Theorem** **3.** *(Semantic Channel Coding Theorem I) For every discrete memoryless channel, the channel capacity*

(25) $$C_{s}=\underset{P(X|Z)}{\sup}\left\{I\left(X;Y\right)-H\left(Z|X\right)+\bar{H_{S}\left(Y\right)}\right\}$$

*has the following property: For any ϵ>0 and $R\leq C_{s}$, there is a block coding strategy such that the maximal probability of semantic error is not greater than ϵ.*

Among them, *X* and *Y* serve as the input and output of the channel, while *Z* is the semantic representation. I(X;Y) denotes the mutual information between *X* and *Y*. H(Z|X) represents the semantic uncertainty associated with the encoding. Additionally, $\bar{H_{S}\left(Y\right)}$ represents the average logical information of the received message.

Based on the theorem presented above, we can see that the semantic capacity may be higher or lower than the physical channel capacity, depending on whether $\bar{H_{S}\left(Y\right)}$ or H(Z|X) is larger. This observation implies that through the utilization of a semantic encoder with minimal semantic ambiguity and a semantic decoder possessing robust inference capabilities or an extensive knowledge base, it is possible to achieve high-rate semantic communication using a low-rate physical channel.

**Figure 5:**
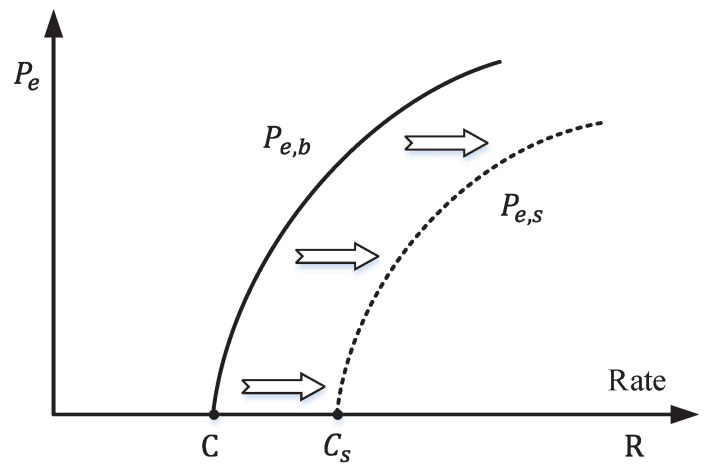
Error probability of the communication system.

It is worth noting that in semantic communication, even when some symbolic errors occur, the underlying semantics may still remain unchanged. In other words, semantic communication may allow a certain level of bit errors, signified by a non-zero BER. Consequently, in cases where the communication system permits a given non-zero BER ($P_{e,b}\geq 0$), the transmission rate *R* can exceed the physical channel capacity *C*. As a response to the second question, Figure 5 shows the error probability of the communication system. It indicates that when R>C, the $P_{e,b}$ becomes greater than zero, while the $P_{e,s}$ (semantic error) may still remain at zero.

**Figure 6:**
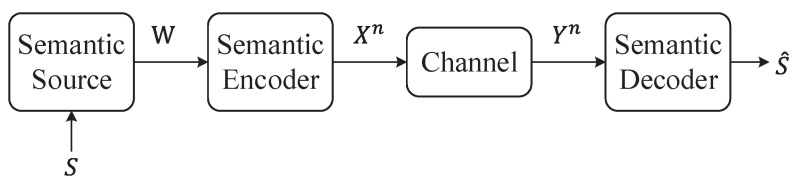
A semantic communication system [56].

Consider a semantic communication system, as illustrated in Figure 6. In this system, a semantic sender aims to reliably transmit a latent semantic message *S* within the message *W* at a rate *R* bits per transmission to a semantic receiver over a noisy physical channel. The source message set is defined as $\left[1:2^{nR}\right]=\left\{1,2,\ldots ,2^{nR}\right\}$, which contains a semantic message subset $\left[1:2^{\left\lceil \alpha nR\right\rceil }\right]=\left\{1,2,\ldots ,2^{\left\lceil \alpha nR\right\rceil }\right\}$, where the coefficient α falls within the range 0≤α<1. Thus, given the semantic mapping $\left[1:2^{nR}\right]\to \left[1:2^{\alpha nR}\right]$ and the discrete memoryless channel p(y|x), Ma et al. [56] define the semantic channel $C_{s}$ as follows:

(26) $$C_{s}=\max_{p(x)}\frac{I(X;Y)}{\alpha }.$$

Moreover, they further give the following theorem concerning semantic channel coding:

**Theorem** **4.** *(Semantic Channel Coding Theorem II) For every bit rate $R<C_{s}=\max_{p(x)}\frac{I(X:Y)}{\alpha }$, there exists a sequence of $\left(2^{nR},n\right)$ codes with an average probability of error $P_{e,s}^{\left(n\right)}$ that tends to zero as n→∞.*

*Conversely, for every sequence of $\left(2^{nR},n\right)$ codes with a probability of error $P_{e,s}^{\left(n\right)}$ that tends to zero as n→∞, the rate must satisfy $R\leq C_{s}=\max_{p(x)}\frac{I(X;Y)}{\alpha }$.*

For the third question, we believe that the semantic channel capacity should be related to the specific task and the background knowledge possessed by both parties—in other words, it is task and goal-oriented. Additionally, we contend that this semantic channel capacity should be dynamic, adapting to the temporal relevance of information. Consequently, establishing a universally applicable expression for the semantic channel capacity becomes a complex undertaking. However, if we try to describe it, we propose that it possesses three distinct characteristics.

1. It takes the form of conditions. These conditions at least shall include tasks, public knowledge, private knowledge, and goals.
2. It has the capability to reflect the temporal value of information. For instance, in situations demanding low latency, messages with slow transmission rates will possess a low information value density.
3. It should encompass the concept of physical channel capacity since the semantic channel does not really exist, and the transmission of symbols must still be achieved through the real physical channel.

## 6. Related Mathematical Theories and Methods

In this section, we introduce some mathematical tools and concepts that are highly relevant to semantic communication. Strictly speaking, these elements do not fall within the scope of semantic information theory, but have been used by several scholars in the study of semantic communication, so we also include them in this review for the sake of comprehensiveness. These include the age of information (AoI), information bottleneck (IB), large language models (LLMs), and joint source channel coding (JSCC). In fact, these methods have already been extensively applied within the realm of semantic communication and have demonstrated their effectiveness. They are expected to become an auxiliary or part of semantic information theory.

### 6.1. Age of Information (AoI)

In the context of semantic communication, the transmitted semantics are usually task-oriented. Many of these tasks require low latency and are sensitive to the freshness of the message. Regardless of how accurately the message can be recovered, if it arrives too late, it can be rendered completely useless, as highlighted by Gündüz in their work [2]. For example, in the realm of autonomous driving, vehicles depend on near-instantaneous data exchange to make split-second decisions, thereby avoiding potential traffic accidents. Similarly, in the context of remote surgery systems, the timely update of surgical tool positions is necessary to ensure the safety and precision of medical procedures. However, this does not imply that the transmitter should update the current state as rapidly as possible. When the network is congested, such a strategy could lead to a monitor receiving delayed updates that were backlogged in the communication system.

Conversely, it is important to note that neither the source statistics nor quality measures change over time in digital communications. Therefore, there exists a necessity to acquire the status of remote sensors or systems. In other words, semantic communication needs to take into account the temporal aspect and its impact on the overall effectiveness of the communication process.

The AoI serves as an end-to-end performance metric, providing a means to quantify the timeliness of a monitor’s knowledge about a particular entity or process [57]. It has the potential to empower semantic communication to measure the freshness of semantics. We will discuss several kinds of AoI and the relationship that exists between semantic communication and the AoI.

**Figure 7:**
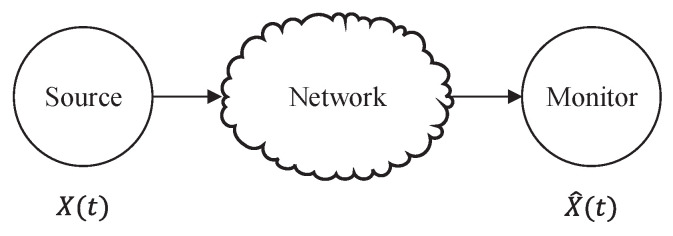
Updates from a source pass through the network to a destination monitor.

As depicted in Figure 7, a source continuously generates new updates to a network that are subsequently delivered to a destination monitor. In this context, the source is represented as a random process denoted as X(t), while the monitor possesses the capability to estimate the current state $\hat{X}\left(t\right)$. Each update packet is associated with a timestamp, denoted as *u*, and its age at time t≥u is defined as t−u. An update is said to be fresh when its timestamp matches the current time *t*, resulting in an age of zero [58]. When the monitor’s most recently received update at time *t* has a timestamp of u(t), the AoI is defined as

(27) ▵(t)=t−u(t).

**Figure 8:**
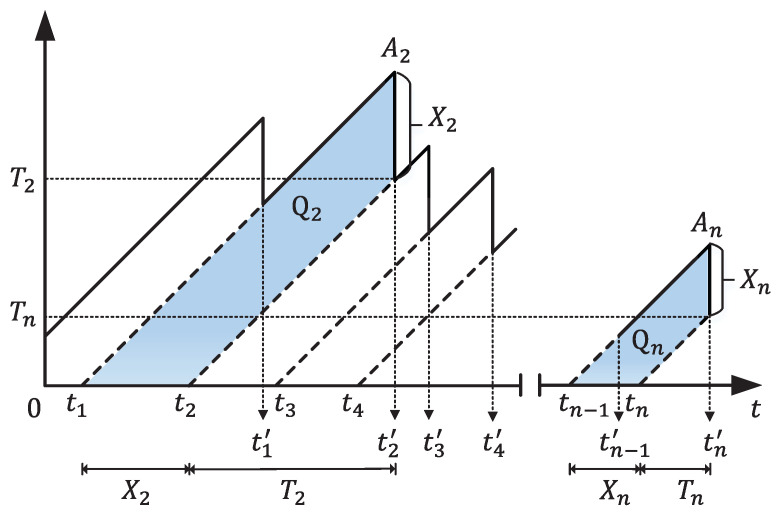
AoI evolution vs. time for *n* update packets [59].

Figure 8 depicts a visualization of the AoI at the monitor over time. In this scenario, the transmitter sends update packets according to a first-come-first-served (FCFS) queuing discipline, allowing only one packet transmission at any given time. Since the monitor sees updates that are delivered at times $t_{j}^{\prime }$ after traveling through the network, its age process ▵t is reset to $▵\left(t_{i}^{\prime }\right)=t_{j}^{\prime }-t_{j}$, which is the age of update *j* when it is delivered. Building upon this foundation, various representations of age can be derived, such as the time average age and the peak age.

For an interval of observation (0,T), the time average age of a status update system is defined as

(28) $${▵}_{T}=\frac{1}{T}\int_{0}^{T}▵\left(t\right)dt.$$

As T→∞, the time average age is calculated as

(29) $$▵=\lim_{T\to \infty }{▵}_{T}=\frac{E[Q_{n}]}{E[T_{n}]}=\frac{E\left[X_{n}T_{n}\right]+E\left[X_{n}^{2}\right]/2}{E[T_{n}]},$$

where $Q_{n}$, $T_{n}$, $X_{n}$ are depicted in Figure 8. $Q_{n}$ corresponds to the dark area in Figure 8. $X_{n}$ represents the interarrival time of the nth update, and $T_{n}$ denotes the corresponding system time.

On the other hand, the difficulty in evaluating $E[X_{n}T_{n}]$ prompted the introduction of the peak age of information (PAoI) [60], an alternative and more manageable metric for age assessment. The PAoI metric is defined as the value of the age achieved immediately before receiving the *n*th update

(30) $$A_{n}=X_{n}+T_{n}.$$

Moreover, the average peak age of a status update system is calculated as

(31) $$A_{T}=\lim_{N\to \infty }\frac{1}{N}\sum_{n=1}^{N(T)}A_{n},$$

where N(T) is the number of samples that are completed by time T. The peak age can be utilized in applications where the worst-case age is of interest or where there is a need to apply a threshold restriction on age.

While the AoI proves valuable in assessing the freshness of information and has seen widespread application across various system contexts employing diverse queuing disciplines, it falls short in effectively capturing the informational content of transmitted packets and the monitor’s current knowledge. In fact, even when the monitor has perfect knowledge of the underlying process in question, the AoI continuously increases over time, leading to unnecessary penalties. This motivated the introduction of a novel metric, known as the age of incorrect information (AoII), which addresses the limitations of the conventional AoI and error penalty functions within the framework of status updates [61]. The AoII is defined as

(32) $$▵\left(t\right)=\left(t-v\left(t\right)\right)\cdot 1\left\{X\left(t\right)\neq \hat{X(t)}\right\},$$

where 1 is the indicator function. When $1\{X\left(t\right)\neq \hat{X(t)}\}=0$, the monitor has the most updated information about X(t) irrespective of when the status update was received. The AoII extends the notion of fresh updates and adequately captures the information content that the updates bring to the monitor.

In a given scenario, a transmitter observes the source process X(t) and sends samples/updates regarding this source over time to a monitor. The primary objective is to reconstruct the original process, X(t), at the monitor utilizing the received samples/updates [62]. In this general setup, the transmitter has two key decisions to make during each time slot: (i) whether to sample X(t) or not, and (ii) whether to transmit the available or newly generated sample/update or not. The real-time compression/transmission has been explored in the literature, with relevant studies such as [63,64].

Moreover, the objective function that takes into account semantic awareness can be formulated as:

(33) $$S=\lim_{T\to \infty }\frac{1}{T}\sum_{t=1}^{T}c\left(t\right),$$

where c(t) represents a cost function that is selected appropriately. For instance, c(t) can be chosen as

(34) $$c\left(t\right)=a\left(t\right)\cdot 1\{X\left(t\right)\neq \hat{X(t)}\},$$

where a(t) is a function of *t*. When one does not take channel or transmission delays into account, the problem described above can be viewed as a rate-distortion problem. Nevertheless, the primary objective in this context is to devise sampling and transmission policies that minimize the average distortion with consideration for the delay sensitivity.

In their work, Gündüz et al. [2] represented three distinct sampling and transmission policies that have subtle connections between the age metrics and semantics of information: AoI-aware, source-aware, semantics-aware sampling and transmission policy. The third policy addresses the limitations of the prior ones by considering not only the state of the source signal but also the status of the reconstructed signals at the monitor. It is expected to be introduced into the semantic communication systems to reflect the impact of time on semantics.

In semantic communication, combining the AoI/AoII and semantic distortion can be a way to evaluate delay-sensitive distortion. In [65], a multi-user uplink non-orthogonal multiple access system is constructed to analyze its transmission performance by harnessing the age of incorrect information. The semantic similarity [40] is adopted as the performance metric and the AoII as the time delay; thus, the instantaneous AoII of the *k*-th user in the scenario can be expressed as

(35) $$\begin{array}{cc} \Psi^{k}\left(t\right) & ≜{▵}_{\mathrm{AoII}}\left(X_{t},{\hat{X}}_{t},t\right) \end{array}$$

(36) $$\begin{array}{cc} \; & =\left(1-\psi_{n}^{k}\left(s,\hat{s}\right)\right)\cdot ▵\left(t\right) \end{array}$$

(37) $$\begin{array}{cc} \; & =\left(1-\frac{B_{\Phi }\left(s\right)\cdot B_{\Phi }{\left(\hat{s}\right)}^{T}}{∥B_{\Phi }\left(s\right)∥∥B_{\Phi }\left(\hat{s}\right)∥}\right)\left(t-u\left(t\right)\right). \end{array}$$

By minimizing the average cost of the system, the optimization problem of multiple users can be solved. This system utilizes the AoII as a metric while simultaneously assessing the semantic similarity and AoI performance.

### 6.2. Information Bottleneck (IB)

Since deep neural networks (DNNs) have demonstrated excellent performance in extracting features and key information, many semantic communication systems use them as a tool adopted in encoders and decoders. IB, an information-theoretic principle, can explore the interplay between the informativeness of extracted features and its effect on communication goals. It offers a novel paradigm for analyzing deep neural networks (DNNs) and tries to shed light on their layered structure, generalization capabilities, and learning dynamics [66], which has been informed by machine learning and information theory [67]. The interaction of IB and DNNs reported in the literature can be divided into two main categories. The first is to use the IB theories in order to analyze DNNs and the other is to use the ideas from IB to improve the DNN-based learning algorithms [68]. Therefore, the IB theory may also be a powerful mathematical tool for the development of semantic communication.

**Figure 9:**
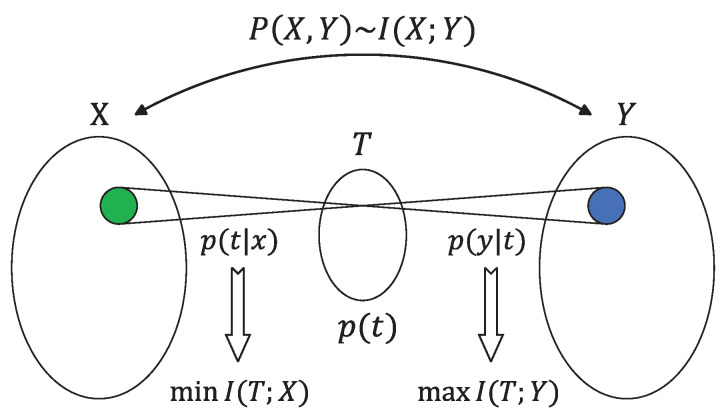
The information between *X* and *Y* is squeezed through the compact “bottleneck” representation, *T*. In particular, under some constraint over the minimal level of relevant information, I(T;Y), one is trying to minimize the compression information, I(T;X).

The IB method was introduced as an information-theoretic principle for extracting relevant information from an input random variable X∈X with respect to an output random variable Y∈Y [69]. In the IB framework, the objective is to compress the information that variable *X* carries about variable *Y* through a compact ’bottleneck’ represented as *T*. Either way, the basic trade-off is between minimizing the compression information and maximizing the relevant information [70]. An illustration of this idea is given in Figure 9.

Given a joint probability distribution p(x,y), the IB optimization problem can be formulated as follows: find *T* such that the mutual information I(T;X) is minimized, subject to the constraint that I(T;Y) does not exceed a predetermined threshold $\hat{D}$. Consequently, it is intuitive to introduce a mathematical function analogous to the rate-distortion function

(38) $$\hat{R}\left(\hat{D}\right)≜\min_{\left\{p\left(t|x\right):I\left(T;Y\right)\leq \hat{D}\right\}}I\left(T;X\right).$$

In a word, $\hat{R}\left(\hat{D}\right)$ represents the minimal achievable compression information, for which the relevant information is above $\hat{R}\left(\hat{D}\right)$. Moreover, the optimal assignment can be determined by minimizing the corresponding functional

(39) L=I(T;X)−βI(T;Y),

where β is the Lagrange multiplier attached to the constrained meaningful information while maintaining the normalization of the mapping p(t;x) for every *x*. At β=0, the quantization is the most sketchy possible; everything is assigned to a single point, while as β→∞, we are pushed toward arbitrarily detailed quantization. By varying the parameter β, one can explore the trade-off between preserving meaningful information and achieving compression at different levels of resolution [71].

When applying the IB method to enhance semantic communication systems, a common approach involves utilizing IB to extract task-related information while eliminating redundant features. Barbarossa et al. [72] presented an approach to semantic communication building on the IB principle. It used the IB as a way to identify relevant information and adapt the IB online, as a function of the wireless channel state, in order to strike an optimal trade-off between the transmit power, the reconstruction accuracy, and the delay. More specifically, Barbarossa et al. considered the case where the transmitted data are corrupted by noise, so that the receiver does not have direct access to *T*, but only to a corrupted version T+η, where η is the channel noise. Then, they redefined the bottleneck optimization problem as

(40) $$\min_{A,M}I\left(X;T\right)+\beta \cdot \mathrm{MSE}\left(Y,\hat{Y}\right),$$

where T=A·X+ξ denotes a linear encoder, including additive noise ξ, while $\hat{Y}=M\cdot \left(T+\eta \right)$ is a linear estimate of *Y*. β is a scalar parameter that allows it to tune the trade-off between complexity and relevant information; small values of β lead to small complexity encoders, but possibly large distortion. Conversely, larger values of β lead to reduced distortion at the expense of increased complexity.

Li et al. [73] used the IB framework to extract distinguishable features in distribution data while keeping their compactness and informativeness. Wei et al. [74] presented a federated semantic learning framework to collaboratively train the semantic-channel encoders of multiple devices with the coordination of a base-station-based semantic-channel decoder. In this approach, the IB is leveraged to drive the loss design by formalizing a rate-distortion trade-off. This trade-off serves to eliminate the redundancies of semantic features while maintaining task-relevant information.

In 2018, Zaslavsky et al. [75] presented empirical evidence that IB may give rise to human-like semantic representations. They conducted research into how human languages categorize colors. Their findings indicated that human languages appear to evolve under pressure to efficiently compress meanings into communication signals by optimizing the IB trade-off between informativeness and complexity. Furthermore, Tucker et al. [76] studied how trading-off three factors—utility, informativeness, and complexity—shapes emergent communication, including compared to human communication. Their study not only shed light on these factors’ impact on communication but also made comparisons to human communication processes.

### 6.3. Joint Source Channel Coding (JSCC)

**Figure 10:**
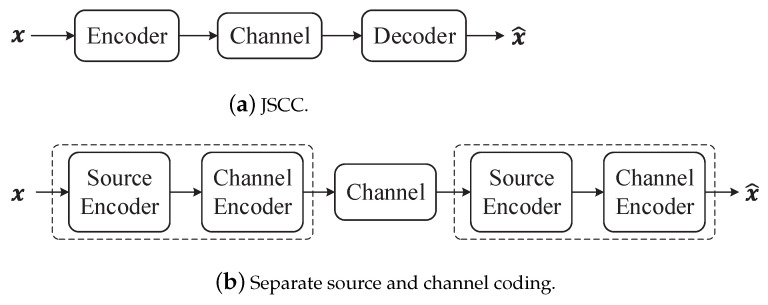
The joint/separate source and channel coding system.

From the perspective of structural design in communication systems, two primary categories emerge: separate source and channel coding, and JSCC. In the case of JSCC, as depicted in Figure 10a, there exists only an encoder and decoder. In this setup, the system optimizes both source coding and channel coding. These coding schemes are integrated into a unified process. Conversely, Figure 10b illustrates the separate source and channel coding system. These two design approaches differ significantly. In Shannon’s theory, the source codes are designed independently to achieve efficient data representation, while the channel codes are designed separately, tailored to the specific characteristics of the channel.

Shannon’s separation theorem is a fundamental result in information theory. It established that separate encoders can achieve the same rates as the joint encoder. Specifically, it tied together the two basic theorems of information theory: data compression and data transmission, as outlined in [32]. The data compression theorem is an outcome of the asymptotic equipartition property (AEP), which shows that there exists a “small” subset (of size $2^{nH}$) of all possible source sequences that contain most of the probability. Consequently, one can represent the source with a small probability of error using an average of *H* bits per symbol. The data transmission theorem, on the other hand, is based on the joint AEP. It capitalizes on the fact that for long block lengths, the output sequence of the channel is very likely to be jointly typical with the input codeword, while any other codeword is jointly typical with probability ∼$2^{-nI}$. As a result, we can employ approximately $2^{nI}$ codewords while still having a negligible probability of error. The source-channel separation theorem shows that one can design the source code and the channel code separately and combine the results to achieve the same optimal performance.

Shannon’s separation theorem proves that the two-step source and channel coding approach is theoretically optimal in the asymptotic limit of infinitely long source and channel blocks. However, in practical applications, it is widely recognized that JSCC surpasses the separate approach in terms of performance especially for limited source and channel coding complexity. Furthermore, JSCC is resilient to variations in channel conditions and does not suffer from abrupt quality degradations, commonly referred to as the “cliff effect” in digital communication systems [77]. Nonetheless, semantic communication is often oriented towards emerging intelligent applications from the Internet-of-Things to autonomous driving and the tactile Internet requires transmission of image/video data under extreme latency, bandwidth, and energy constraints. This precludes computationally demanding long-block-length source and channel coding techniques. Therefore, JSCC may represent a potential trend in semantic communication systems.

In recent years, semantic communication systems employing JSCC have demonstrated superior performance across various domains, surpassing separate design approaches and offering new potential applications and advantages [78,79]. In 2019, a noteworthy development known as Deep JSCC [77] introduced encoder and decoder functions parameterized by two convolutional neural networks, trained jointly. It is designed for the transmission of images. The results show that the Deep JSCC scheme outperforms digital transmission concatenating JPEG or JPEG2000 compression with a capacity-achieving channel code at low SNR and channel bandwidth values in the presence of additive white Gaussian noise (AWGN). Building upon this foundation, Deep JSCC-f [80] investigated how noiseless or noisy channel output feedback can be incorporated into the Deep JSCC to improve the reconstruction quality at the receiver. With the advancement of DL, more novel neural network structures have been introduced into semantic communication [81]. These structures replace autoencoders to achieve the semantic transmission of images, resulting in improved performance.

JSCC also shows excellent performance across various carriers. Xie et al. [40] proposed DeepSC, a DL-based semantic communication system designed for text transmission. In comparison to traditional communication systems that do not account for semantic information exchange, DeepSC exhibits remarkable robustness to channel variations and superior performance, particularly in low SNR conditions. For speech signals, DeepSC-S [82] was designed, which outperforms traditional communications in both cases in terms of the speech signals metrics, such as the signal-to-distortion ratio and the perceptual evaluation of speech distortion. Lastly, MU-DeepSC [83] represents a multi-user semantic communication system for transmitting multimodal data. Its transceiver is ingeniously designed and optimized to capture features from correlated multimodal data, facilitating task-oriented transmission. These recent advancements highlight the growing potential and versatility of JSCC in semantic communication systems across a range of data types and applications.

### 6.4. Large Language Models (LLMs)

In a semantic communication system, the knowledge base plays a crucial role in enabling the semantic encoder and decoder to comprehend and infer semantic information. In general, knowledge includes public knowledge and private knowledge. The former is shared by all communication participants, while the latter is unique to a user. In fact, the knowledge base is a key feature that distinguishes semantic communication from conventional communication systems. However, the representation and updating of knowledge is a challenging task, which is also one of the factors that make semantic communication difficult to mathematically model. In recent years, LLMs have developed rapidly and have shown great potential in intelligent tasks [84]. In this subsection, we will introduce the feasibility of introducing LLMs into semantic communication. We believe that LLMs may play a potential role in knowledge bases for semantic communication.

Language models (LMs) are computational models that have the capacity to understand and generate human language [85]. These models possess the transformative ability to predict the likelihood of word sequences or to generate new text based on a given input [86]. In the context of a sequence denoted as *X*, LM tasks aim to predict the next token, denoted as *y*. The model is trained by maximizing the probability of the given token sequence conditioned on the context, i.e., $P\left(y|X\right)=P\left(y|x_{1},x_{2},\ldots ,x_{n-1}\right)$, where $x_{1},x_{2},\ldots ,x_{n-1}$ are the tokens in the context sequence, and *n* is the current position. Utilizing the chain rule, the conditional probability can be decomposed into a product of probabilities at each position:

(41) $$p\left(y|X\right)=\prod_{n=1}^{N}P\left(y_{t}|x_{1},x_{2},\ldots ,x_{t-1}\right),$$

where *N* represents the length of the sequence. Consequently, the model predicts each token at each position in an autoregressive manner, ultimately generating a complete text sequence.

LLMs are advanced language models with massive parameter sizes and exceptional learning capabilities. The core module behind many LLMs, such as GPT-3 [87], InstructGPT [88], and GPT-4, is the self-attention module in the Transformer [89] architecture. This self-attention module serves as the foundational building block for various language modeling tasks.

A fundamental characteristic of LLMs lies in their capability for in-context learning. This means the model is trained to generate text based on a provided context or prompt. This capability empowers LLMs to produce responses that are not only more coherent but also contextually relevant, making them well-suited for interactive and conversational applications. Another crucial aspect of LLMs is reinforcement learning from human feedback (RLHF) [90]. This technique involves fine-tuning the model by using human-generated responses as rewards, enabling the model to learn from its mistakes and to progressively enhance its performance over time.

**Figure 11:**
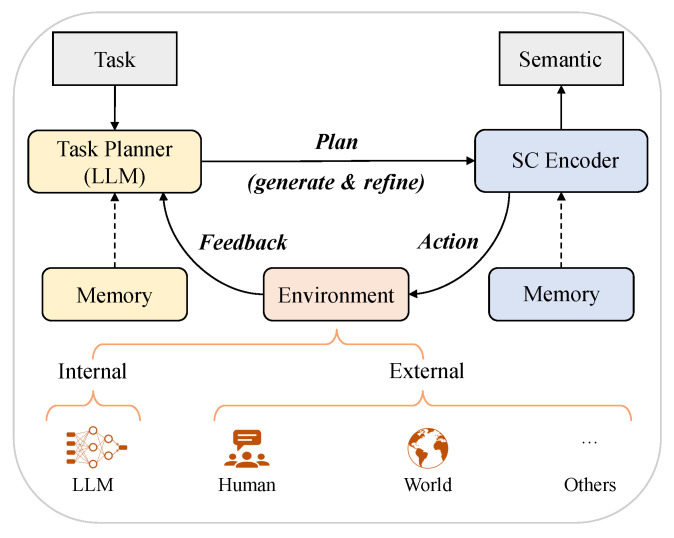
An illustration of the formulation for prompt-based planning by LLMs for semantic communication (transmitter) [91].

Since LLMs use a large amount of data, parameters, and even human feedback during training, they have a perception and understanding of the world, which can be called the knowledge base to a certain extent. On the other hand, due to the excellent performance and capabilities of LLMs, they have the potential to be applied in a variety of intelligent tasks. Zhao et al. [91] formulated the general planning paradigm of LLMs for solving complex tasks. We think it provides insights into semantic communication based on LLMs, which is illustrated in Figure 11.

Figure 11 illustrates the process by which LLMs help the transmitter’s semantic encoder extract semantics. In this paradigm, there are typically three components: a task planner, a semantic encoder, and an environment. Specifically, the task planner, which is played by LLMs, aims to generate the whole plan to solve a target task-oriented communication. The plan can be presented in various forms, e.g., a visual question answering task [83] or a text transmission task [40]. Then, the semantic encoder is responsible for executing the actions in the plan and generating semantics. It can be implemented by models like LLMs for textual tasks. Furthermore, the environment refers to where the semantic encoder generates the semantics, which can be set differently according to specific tasks. It provides feedback about the execution result of the action to the task planner, either in the form of natural language or from other multimodal signals.

The knowledge base is a significant feature and a crucial component of semantic communication, directly impacting its performance. LMs contain quite a substantial amount of knowledge and have the potential to serve as a knowledge base for semantic communication. Jiang et al. [92] proposed the use of LMs as knowledge bases in semantic communication, representing three LM-based knowledge bases in semantic communication models: (1) GPT-Based: utilizing ChatGPT as the knowledge base for textual data, it enables the extraction of key content from the input text, tailored to user requirements. (2) SAM-Based: employing SAM [93] as a knowledge base for image-related semantic communication, which could be capable of segmenting various objects within an image and recognizing their respective categories and relationships. (3) WavLM-Based: utilizing WavLM [94] as a knowledge base for semantic communication systems involving audio. This includes applications such as automatic speech recognition, speaker identification, and speech separation.

## 7. Discussion

Although this review extensively surveys and analyzes various works related to semantic information theory, delving into aspects such as the semantic entropy, semantic channel capacity, and semantic rate-distortion, along with the methodologies adopted by scholars in semantic communication research, it is important to note that both semantic information theory and semantic communication are still in their initial stages of development. Currently, a unified consensus and a comprehensive theoretical framework have not yet been established. Practical and effective applications in these fields remain a distant prospect at this point in time.

On the other hand, we firmly believe that semantic information theory is not intended to replace classical information theory; rather, it serves as an extension of classical information theory at the semantic level by inputting some new elements or factors from the viewpoints of applications. Nevertheless, there are still some significant differences between them, which is exactly the problem that semantic information theory aims to address. Distinguishing semantic information theory from classical information theory reveals several notable disparities:

- Whether a message is true or not is irrelevant in classical information theory.
- Whether a message is related to the task/time is immaterial in classical information theory.
- Whether a message can effectively convey meaning is not a concern of classical information theory.

However, these differences or concerns about semantic communication are challenging issues at a theoretical level. In fact, the development of semantic information theory is in its infancy, with a large number of open problems that have not yet been solved, such as:

1. The Role of Semantics in Data Compression and Reliable Communication: How can semantics contribute to data compression and enhance the reliability of communication to some targets in applications?
2. Relationship Between Semantic and Engineering Coding: What is the interplay between semantic coding/decoding techniques and conventional engineering coding/coding problems?
3. Fundamental Limits of Semantic Communication: Are there established limits or boundaries in semantic coding?
4. Enhancing Efficiency and Reliability in Semantic Communication: What factors should be taken into account to improve efficiency and reliability in semantic communication?
5. Principles for DL-Based Semantic Communication: How should we architect the framework of a semantic communication system rooted in DL, and what theoretical guidance exists?
6. Capacity of Semantic-Aware Networks: What is the capacity of a semantic network, and how can we evaluate the performance limits of a semantic transmission network?
7. The effect of communication networking topologies: What is the effect of communication networking topologies on semantic communication? For example, the key feature of semantic communication over ad hoc networks [95], relay networks [96,97], multiple access/broadcast networks [98], as well as distributed free cell networks [99], also need to be investigated in the near future.

Currently, solving these theoretical challenges is formidable, but necessary. The resolution of these issues is pivotal for semantic information theory to achieve the same depth and solidity as classical information theory. Beyond the theoretical realm, semantic communication systems present an array of open challenges:

1. Scheduling and Energy Optimization: Delving into scheduling and resource allocation policies within semantic communication, with a concentrated effort on optimizing energy utilization.
2. Complexity of Semantic-Enabled Networks: Semantic-enabled networks face high complexity due to the need to share knowledge with users. This necessitates a framework for evaluating the complexity and necessity of semantic communication networks.
3. Multi-criteria Optimization: Developing strategies for semantic communication in scenarios where multiple tasks and objectives coexist.
4. Knowledge Updates Tracking: Recognizing that knowledge can evolve over time within semantic networks.
5. Applications: Identifying specific use cases and applications that align with semantic communication systems.
6. Performance Metrics: Defining comprehensive performance metrics for assessing the effectiveness and efficiency of semantic communication systems.

In this section, we introduce open issues within semantic information theory and semantic communication, with the aim of stimulating further exploration and fostering meaningful discussions among researchers.

## 8. Concluding Remarks

Semantic communication, as an innovative communication structure, has revolutionized the traditional data transmission paradigm and has the potential to provide fresh insights into large-scale intelligent processing services. Nevertheless, it is important to note that the field of semantic communication is still in its infancy, offering abundant opportunities for further exploration and research.

This article systematically summarized the development of semantic information theory, encompassing a comprehensive review of related advancements. Beginning with an exploration of semantic entropy, we further introduced statistical probability, logical probability, semantic rate-distortion, semantic encoding, semantic noise, and semantic channel capacity. Moreover, the article presented a structured exposition of the mathematical theories and methodologies relevant to semantic communication, including concepts like the AoI, IB, and JSCC.

In addition, we investigated the prevalent challenges and open problems within the realm of semantic information theory and semantic communication. We believe that this article will make a meaningful contribution to the establishment of semantic information theory and the rapid evolution of semantic communication.

## Data Availability

Not applicable.

## References

[1] Letaief K.B., Chen W., Shi Y., Zhang J., Zhang Y.J.A. (2019). The roadmap to 6G: AI empowered wireless networks. IEEE Commun. Mag..

[2] Gündüz D., Qin Z., Aguerri I.E., Dhillon H.S., Yang Z., Yener A., Wong K.K., Chae C.B. (2022). Beyond transmitting bits: Context, semantics, and task-oriented communications. IEEE J. Sel. Areas Commun..

[3] Yang W., Du H., Liew Z.Q., Lim W.Y.B., Xiong Z., Niyato D., Chi X., Shen X.S., Miao C. (2022). Semantic communications for future internet: Fundamentals, applications, and challenges. IEEE Commun. Surv. Tutor..

[4] Shannon C.E., Weaver W. (1949). The Mathematical Theory of Communication.

[5] Qin Z., Tao X., Lu J., Tong W., Li G.Y. (2021). Semantic communications: Principles and challenges. arXiv.

[6] Strinati E.C., Barbarossa S. (2021). 6G networks: Beyond Shannon towards semantic and goal-oriented communications. Comput. Netw..

[7] Shi G., Xiao Y., Li Y., Xie X. (2021). From semantic communication to semantic-aware networking: Model, architecture, and open problems. IEEE Commun. Mag..

[8] Kountouris M., Pappas N. (2021). Semantics-empowered communication for networked intelligent systems. IEEE Commun. Mag..

[9] Kalfa M., Gok M., Atalik A., Tegin B., Duman T.M., Arikan O. (2021). Towards goal-oriented semantic signal processing: Applications and future challenges. Digit. Signal Process..

[10] Lan Q., Wen D., Zhang Z., Zeng Q., Chen X., Popovski P., Huang K. (2021). What is semantic communication? A view on conveying meaning in the era of machine intelligence. J. Commun. Inf. Netw..

[11] Uysal E., Kaya O., Ephremides A., Gross J., Codreanu M., Popovski P., Assaad M., Liva G., Munari A., Soret B. (2022). Semantic communications in networked systems: A data significance perspective. IEEE Netw..

[12] Zhang P., Xu W., Gao H., Niu K., Xu X., Qin X., Yuan C., Qin Z., Zhao H., Wei J. (2022). Toward wisdom-evolutionary and primitive-concise 6G: A new paradigm of semantic communication networks. Engineering.

[13] Shi Y., Zhou Y., Wen D., Wu Y., Jiang C., Letaief K.B. (2023). Task-oriented communications for 6g: Vision, principles, and technologies. arXiv.

[14] Shannon C.E. (1948). A mathematical theory of communication. Bell Syst. Tech. J..

[15] Bao J., Basu P., Dean M., Partridge C., Swami A., Leland W., Hendler J.A. (2011). Towards a theory of semantic communication. Proceedings of the 2011 IEEE Network Science Workshop.

[16] Iyer S., Khanai R., Torse D., Pandya R.J., Rabie K.M., Pai K., Khan W.U., Fadlullah Z. (2023). A survey on semantic communications for intelligent wireless networks. Wirel. Pers. Commun..

[17] Carnap R. (1950). Logical Foundations of Probability.

[18] Carnap R., Bar-Hillel Y. (1952). An Outline of a Theory of Semantic Information.

[19] Floridi L. (2004). Outline of a theory of strongly semantic information. Minds Mach..

[20] D’Alfonso S. (2011). On quantifying semantic information. Information.

[21] Basu P., Bao J., Dean M., Hendler J. (2014). Preserving quality of information by using semantic relationships. Pervasive Mob. Comput..

[22] Chattopadhyay A., Haeffele B.D., Geman D., Vidal R. (2020). Quantifying Task Complexity through Generalized Information Measures. https://openreview.net/forum?id=vcKVhY7AZqK.

[23] Melamed I.D. Measuring semantic entropy. Proceedings of the Tagging Text with Lexical Semantics: Why, What, and How?.

[24] Liu X., Jia W., Liu W., Pedrycz W. (2019). AFSSE: An interpretable classifier with axiomatic fuzzy set and semantic entropy. IEEE Trans. Fuzzy Syst..

[25] De Luca A., Termini S. (1993). A definition of a nonprobabilistic entropy in the setting of fuzzy sets theory. Readings in Fuzzy Sets for Intelligent Systems.

[26] Choi J., Loke S.W., Park J. (2022). A unified view on semantic information and communication: A probabilistic logic approach. Proceedings of the 2022 IEEE International Conference on Communications Workshops (ICC Workshops).

[27] Xin G., Fan P. (2022). EXK-SC: A semantic communication model based on information framework expansion and knowledge collision. Entropy.

[28] Xin G., Zhu Z., Fan P. (2023). Information Framework Expansion Meets Knowledge Collision for Semantic Communications. Proceedings of the ICC 2023-IEEE International Conference on Communications.

[29] Kolchinsky A., Wolpert D.H. (2018). Semantic information, autonomous agency and non-equilibrium statistical physics. Interface Focus.

[30] Venhuizen N.J., Crocker M.W., Brouwer H. (2019). Semantic entropy in language comprehension. Entropy.

[31] Lu C. (2018). From Bayesian inference to logical Bayesian inference: A new mathematical frame for semantic communication and machine learning. Proceedings of the Intelligence Science II: Third IFIP TC 12 International Conference, ICIS 2018.

[32] Cover T.M., Thomas J.A. (1991). Elements of Information Theory.

[33] Johnson J., Alahi A., Fei-Fei L. (2016). Perceptual losses for real-time style transfer and super-resolution. Proceedings of the Computer Vision–ECCV 2016: 14th European Conference.

[34] Zhang R., Isola P., Efros A.A., Shechtman E., Wang O. The unreasonable effectiveness of deep features as a perceptual metric. Proceedings of the IEEE Conference on Computer Vision and Pattern Recognition.

[35] Wang J., Song Y., Leung T., Rosenberg C., Wang J., Philbin J., Chen B., Wu Y. Learning fine-grained image similarity with deep ranking. Proceedings of the IEEE Conference on Computer Vision and Pattern Recognition.

[36] Zhu T., Peng B., Liang J., Han T., Wan H., Fu J., Chen J. (2023). How to Evaluate Semantic Communications for Images with ViTScore Metric?. arXiv.

[37] Farsad N., Rao M., Goldsmith A. (2018). Deep learning for joint source-channel coding of text. Proceedings of the 2018 IEEE International Conference on Acoustics, Speech and Signal Processing (ICASSP).

[38] Güler B., Yener A., Swami A. (2018). The semantic communication game. IEEE Trans. Cogn. Commun. Netw..

[39] Papineni K., Roukos S., Ward T., Zhu W.J. Bleu: A method for automatic evaluation of machine translation. Proceedings of the 40th Annual Meeting of the Association for Computational Linguistics.

[40] Xie H., Qin Z., Li G.Y., Juang B.H. (2021). Deep learning enabled semantic communication systems. IEEE Trans. Signal Process..

[41] Devlin J., Chang M.W., Lee K., Toutanova K. (2018). Bert: Pre-training of deep bidirectional transformers for language understanding. arXiv.

[42] Rix A.W., Beerends J.G., Hollier M.P., Hekstra A.P. (2001). Perceptual evaluation of speech quality (PESQ)—A new method for speech quality assessment of telephone networks and codecs. Proceedings of the 2001 IEEE International Conference on Acoustics, Speech, and Signal Processing.

[43] Taal C.H., Hendriks R.C., Heusdens R., Jensen J. (2011). An algorithm for intelligibility prediction of time–frequency weighted noisy speech. IEEE Trans. Audio Speech Lang. Process..

[44] Bińkowski M., Donahue J., Dieleman S., Clark A., Elsen E., Casagrande N., Cobo L.C., Simonyan K. (2019). High fidelity speech synthesis with adversarial networks. arXiv.

[45] Liu J., Zhang W., Poor H.V. (2021). A rate-distortion framework for characterizing semantic information. Proceedings of the 2021 IEEE International Symposium on Information Theory (ISIT).

[46] Guo T., Wang Y., Han J., Wu H., Bai B., Han W. (2022). Semantic compression with side information: A rate-distortion perspective. arXiv.

[47] Stavrou P.A., Kountouris M. (2022). A rate distortion approach to goal-oriented communication. Proceedings of the 2022 IEEE International Symposium on Information Theory (ISIT).

[48] Shao Y., Cao Q., Gunduz D. (2022). A theory of semantic communication. arXiv.

[49] Agheli P., Pappas N., Kountouris M. (2022). Semantic Source Coding for Two Users with Heterogeneous Goals. Proceedings of the GLOBECOM 2022–2022 IEEE Global Communications Conference.

[50] Xiao Y., Zhang X., Li Y., Shi G., Başar T. (2022). Rate-distortion theory for strategic semantic communication. Proceedings of the 2022 IEEE Information Theory Workshop (ITW).

[51] Tang J., Yang Q., Zhang Z. (2023). Information-Theoretic Limits on Compression of Semantic Information. arXiv.

[52] Verdu S. (1998). Fifty years of Shannon theory. IEEE Trans. Inf. Theory.

[53] Hu Q., Zhang G., Qin Z., Cai Y., Yu G., Li G.Y. (2023). Robust semantic communications with masked VQ-VAE enabled codebook. IEEE Trans. Wirel. Commun..

[54] Goodfellow I.J., Shlens J., Szegedy C. (2014). Explaining and harnessing adversarial examples. arXiv.

[55] Okamoto T. (2016). A unified paradigm of organized complexity and semantic information theory. arXiv.

[56] Ma S., Wu Y., Qi H., Li H., Shi G., Liang Y., Al-Dhahir N. (2023). A Theory for Semantic Communications. arXiv.

[57] Kosta A., Pappas N., Angelakis V. (2017). Age of information: A new concept, metric, and tool. Found. Trends Netw..

[58] Yates R.D., Sun Y., Brown D.R., Kaul S.K., Modiano E., Ulukus S. (2021). Age of information: An introduction and survey. IEEE J. Sel. Areas Commun..

[59] Abd-Elmagid M.A., Pappas N., Dhillon H.S. (2019). On the role of age of information in the Internet of Things. IEEE Commun. Mag..

[60] Costa M., Codreanu M., Ephremides A. (2014). Age of information with packet management. Proceedings of the 2014 IEEE International Symposium on Information Theory.

[61] Maatouk A., Kriouile S., Assaad M., Ephremides A. (2020). The age of incorrect information: A new performance metric for status updates. IEEE/ACM Trans. Netw..

[62] Sun Y., Polyanskiy Y., Uysal E. (2019). Sampling of the Wiener process for remote estimation over a channel with random delay. IEEE Trans. Inf. Theory.

[63] Witsenhausen H.S. (1979). On the Structure of Real-Time Source Coders. Bell Syst. Tech. J..

[64] Mahajan A., Teneketzis D. (2009). Optimal design of sequential real-time communication systems. IEEE Trans. Inf. Theory.

[65] Chen J., Wang J., Jiang C., Wang J. (2023). Age of Incorrect Information in Semantic Communications for NOMA Aided XR Applications. IEEE J. Sel. Top. Signal Process..

[66] Goldfeld Z., Polyanskiy Y. (2020). The information bottleneck problem and its applications in machine learning. IEEE J. Sel. Areas Inf. Theory.

[67] Shao J., Mao Y., Zhang J. (2021). Learning task-oriented communication for edge inference: An information bottleneck approach. IEEE J. Sel. Areas Commun..

[68] Hafez-Kolahi H., Kasaei S. (2019). Information bottleneck and its applications in deep learning. arXiv.

[69] Tishby N., Zaslavsky N. (2015). Deep learning and the information bottleneck principle. Proceedings of the 2015 IEEE Information Theory Workshop (ITW).

[70] Slonim N. (2002). The Information Bottleneck: Theory and Applications. Ph.D. Thesis.

[71] Tishby N., Pereira F.C., Bialek W. (2000). The information bottleneck method. arXiv.

[72] Barbarossa S., Comminiello D., Grassucci E., Pezone F., Sardellitti S., Di Lorenzo P. (2023). Semantic communications based on adaptive generative models and information bottleneck. arXiv.

[73] Li H., Yu W., He H., Shao J., Song S., Zhang J., Letaief K.B. (2023). Task-Oriented Communication with Out-of-Distribution Detection: An Information Bottleneck Framework. arXiv.

[74] Wei H., Ni W., Xu W., Wang F., Niyato D., Zhang P. (2023). Federated Semantic Learning Driven by Information Bottleneck for Task-Oriented Communications. IEEE Commun. Lett..

[75] Zaslavsky N., Kemp C., Regier T., Tishby N. (2018). Efficient human-like semantic representations via the information bottleneck principle. arXiv.

[76] Tucker M., Shah J., Levy R., Zaslavsky N. (2022). Towards human-agent communication via the information bottleneck principle. arXiv.

[77] Bourtsoulatze E., Kurka D.B., Gündüz D. (2019). Deep joint source-channel coding for wireless image transmission. IEEE Trans. Cogn. Commun. Netw..

[78] Zhang H., Shao S., Tao M., Bi X., Letaief K.B. (2022). Deep learning-enabled semantic communication systems with task-unaware transmitter and dynamic data. IEEE J. Sel. Areas Commun..

[79] Xie H., Qin Z., Tao X., Letaief K.B. (2022). Task-oriented multi-user semantic communications. IEEE J. Sel. Areas Commun..

[80] Kurka D.B., Gündüz D. (2020). DeepJSCC-f: Deep joint source-channel coding of images with feedback. IEEE J. Sel. Areas Inf. Theory.

[81] Kang J., Du H., Li Z., Xiong Z., Ma S., Niyato D., Li Y. (2022). Personalized saliency in task-oriented semantic communications: Image transmission and performance analysis. IEEE J. Sel. Areas Commun..

[82] Weng Z., Qin Z. (2021). Semantic communication systems for speech transmission. IEEE J. Sel. Areas Commun..

[83] Xie H., Qin Z., Li G.Y. (2021). Task-oriented multi-user semantic communications for VQA. IEEE Wirel. Commun. Lett..

[84] Huang J., Chang K.C.C. (2022). Towards reasoning in large language models: A survey. arXiv.

[85] Min B., Ross H., Sulem E., Veyseh A.P.B., Nguyen T.H., Sainz O., Agirre E., Heintz I., Roth D. (2023). Recent advances in natural language processing via large pre-trained language models: A survey. ACM Comput. Surv..

[86] Chang Y., Wang X., Wang J., Wu Y., Zhu K., Chen H., Yang L., Yi X., Wang C., Wang Y. (2023). A survey on evaluation of large language models. arXiv.

[87] Floridi L., Chiriatti M. (2020). GPT-3: Its nature, scope, limits, and consequences. Minds Mach..

[88] Ouyang L., Wu J., Jiang X., Almeida D., Wainwright C., Mishkin P., Zhang C., Agarwal S., Slama K., Ray A. (2022). Training language models to follow instructions with human feedback. Adv. Neural Inf. Process. Syst..

[89] Vaswani A., Shazeer N., Parmar N., Uszkoreit J., Jones L., Gomez A.N., Kaiser Ł., Polosukhin I. (2017). Attention is all you need. Adv. Neural Inf. Process. Syst..

[90] Christiano P.F., Leike J., Brown T., Martic M., Legg S., Amodei D. (2017). Deep reinforcement learning from human preferences. Adv. Neural Inf. Process. Syst..

[91] Zhao W.X., Zhou K., Li J., Tang T., Wang X., Hou Y., Min Y., Zhang B., Zhang J., Dong Z. (2023). A survey of large language models. arXiv.

[92] Jiang F., Peng Y., Dong L., Wang K., Yang K., Pan C., You X. (2023). Large AI Model-Based Semantic Communications. arXiv.

[93] Kirillov A., Mintun E., Ravi N., Mao H., Rolland C., Gustafson L., Xiao T., Whitehead S., Berg A.C., Lo W.Y. (2023). Segment anything. arXiv.

[94] Chen S., Wang C., Chen Z., Wu Y., Liu S., Chen Z., Li J., Kanda N., Yoshioka T., Xiao X. (2022). Wavlm: Large-scale self-supervised pre-training for full stack speech processing. IEEE J. Sel. Top. Signal Process..

[95] Ramanathan R., Redi J. (2002). A brief overview of ad hoc networks: Challenges and directions. IEEE Commun. Mag..

[96] Xiong K., Fan P., Xu Z., Yang H.C., Letaief K.B. (2014). Optimal cooperative beamforming design for MIMO decode-and-forward relay channels. IEEE Trans. Signal Process..

[97] Lin W., Yan Y., Li L., Han Z., Matsumoto T. (2024). Semantic-Forward Relaying: A Novel Framework Towards 6G Cooperative Communications. IEEE Commun. Lett..

[98] Fan P., Zhi C., Wei C., Letaief K.B. (2009). Reliable relay assisted wireless multicast using network coding. IEEE J. Sel. Areas Commun..

[99] Buzzi S., D’Andrea C., Zappone A., D’Elia C. (2019). User-centric 5G cellular networks: Resource allocation and comparison with the cell-free massive MIMO approach. IEEE Trans. Wirel. Commun..

